# A Case of Septic Arthritis of the Wrist due to *Finegoldia magna*

**DOI:** 10.1155/2014/793053

**Published:** 2014-04-13

**Authors:** Camelia Arsene, Abhijit Saste, Manya Somiah, Janee Mestrovich, Gregory Berger

**Affiliations:** ^1^Department of Medicine, Sinai-Grace Hospital, Detroit Medical Center/Wayne State School of Medicine, 4th Floor, 6071 West Outer Drive, Detroit, MI 48235, USA; ^2^Department of Emergency Medicine, Detroit Receiving Hospital/Detroit Medical Center, 4201 Street Antoine, Suite 3R, Detroit, MI 48201, USA

## Abstract

*Finegoldia magna* (*F. magna*) has been described as one of the most frequent pathogens in the etiology of postoperative and prosthetic implant associated septic arthritis. In this report, we document our first experience with septic arthritis of the wrist caused by *F. magna* occurring in a joint with primary disease from prior trauma.

## 1. Introduction

In this report we document our first experience with chronic septic arthritis of the wrist caused by* Finegoldia magna* (*F. magna*) occurring in a joint with preexisting secondary osteoarthritis from prior remote trauma.

## 2. Case Presentation

A 60-year-old African American male presented with progressively increasing erythema and pain and swelling of the right wrist associated with restricted wrist movement. His symptoms began three months prior to presentation when he injured the same wrist while fixing a used tire. He abraded his wrist against the dirty tire and 2 weeks thereafter his wrist started to progressively swell. A week prior to presentation yellowish pus started emanating from the wrist wound. He tried soaking his wrist in warm water and applied topical antibiotic cream without relief. He had a long-standing history of chronic right wrist pain following a closed fracture of his right wrist from a motor vehicle accident sustained 9 years prior to presentation, with preexisting radiographic evidence of wrist joint erosion with cystic changes in the distal radius, ulna, and carpal bones together with joint space narrowing suggesting secondary posttraumatic osteoarthritis. The patient also had a history of polysubstance abuse which included intravenous (IV) heroin use and alcohol abuse. This may have rendered him immunosuppressed and therefore susceptible to this invasive and uncommon infectious agent.

Physical examination findings included an elevated blood pressure of 156/83 and a heart rate of 115/min likely reactive to pain. He was afebrile. Examination of the right hand and wrist revealed an erythematous, fluctuant mass, 3 × 3 cm in size with an open wound on the radial and volar aspect of the wrist draining a purulent foul-smelling discharge.

**Figure 1:**
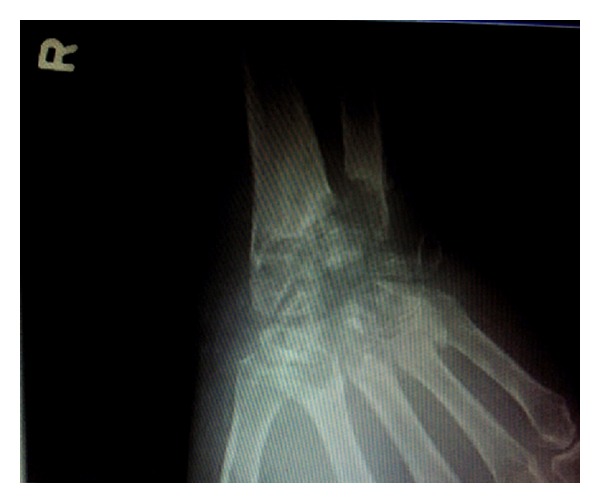
X-ray anteroposterior view of wrist joint: septic arthritis of distal radius/ulna, carpal bones, and bases of metacarpals.

Wrist radiography at admission showed extensive cystic changes involving the distal radius and ulna. There was almost complete loss of the distal ulna and loss of several carpal bones. Cystic erosions extended into the bases of metacarpals. Proximally the radius and ulna appeared normal. These changes were compatible with extensive destructive arthritic disease, the foremost consideration for which being pyogenic arthritis. There was no evidence of soft tissue gas (Figure 1).

**Figure 2:**
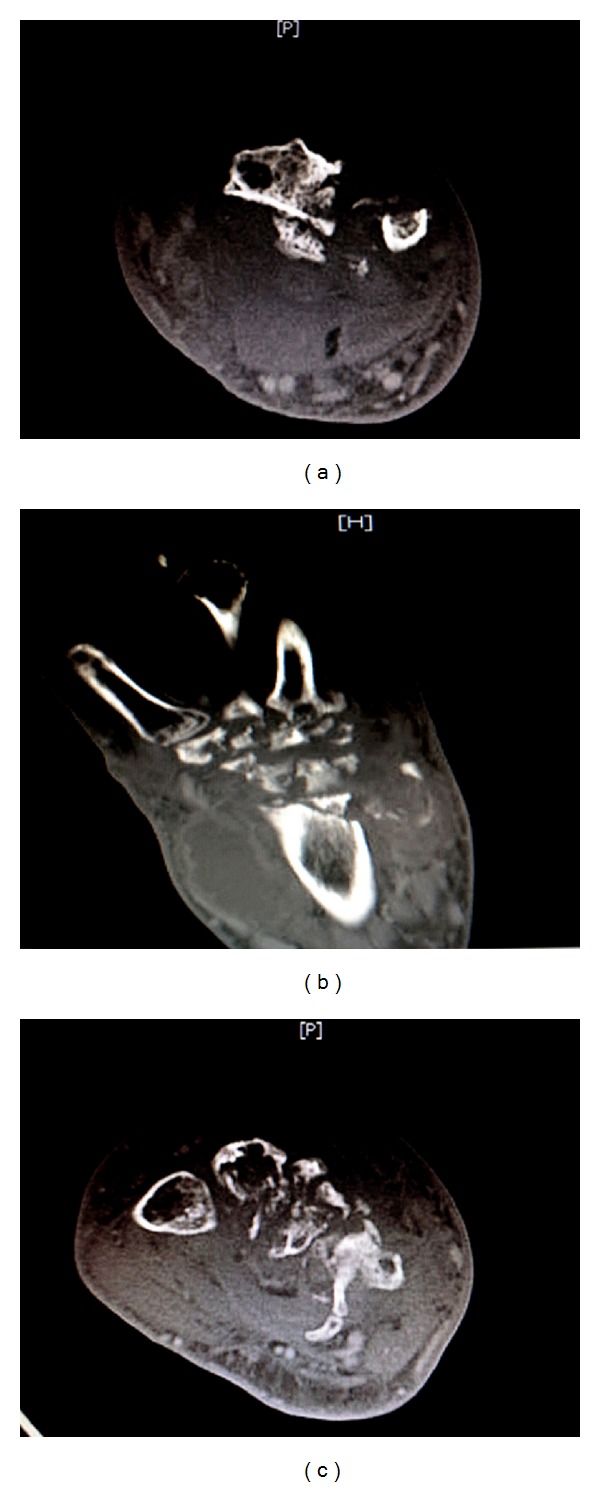
(a) CT wrist transverse section: destructive changes involving distal radius and ulna associated with septated fluid collections with enhancing borders. (b) CT wrist longitudinal section: destructive changes seen in distal radius/ulna, carpal bones, and bases of metacarpals. Pyogenic collections seen surrounding the wrist joint. (c) CT wrist transverse section: destructive changes seen involving the bases of metacarpals in septic arthritis with surrounding pyogenic collections.

Computed tomography (CT) scan of the wrist showed destructive changes involving the base of the second, third, fourth, and fifth metacarpals. More proximally destructive changes extended to involve all carpal bones. There was complete loss of normal anatomic bony relationships. Intercarpal, carpometacarpal, radiocarpal, and ulnocarpal joints were all lost. Similar extensive permeative and destructive changes were seen in the distal radius and ulna. Even more proximally were subtle permeative changes involving the cortex. Associated with this extensive destructive change in the bone were also seen irregularly shaped, thick-walled, septated fluid collections with enhancing borders. These collections were largest on the volar aspect of the wrist and proximal forearm along the radial border. There was no clear compartmentalization. Edema was seen in the subcutaneous tissue. These changes reflected an extensive pyogenic joint with osteomyelitis (Figures 2(a), 2(b), and 2(c)).

Laboratory values showed a leukocytosis with mild left shift and 7% bandemia, C-reactive protein elevation of 34.80 mg/L, and creatinine of 1.5 mg/dL.

The patient was started on empiric IV Vancomycin 1 g Q12H and IV Ceftriaxone 1gm Q24H at the time of presentation. Blood cultures were sent prior to the administration of antibiotics. CT guided aspiration was performed thereafter and the aspirate was sent for microbiologic analysis. The patient also underwent an open arthrotomy with incision and drainage of frank pus from the right wrist after his wrist swelling persisted status after CT guided aspiration. The patient was on empiric antibiotic therapy prior to collecting intraoperative microbiological samples for analysis. Anaerobic blood agar plates (AnBAP) Becton Dickinson, Kanamycin-vancomycin laked blood agar (KV-BAP) Becton Dickinson, Colistin-nalidixic acid blood agar (CNA-BAP) Becton Dickinson, and prereduced chopped meat broth (CMG) Becton Dickinson showed growth of bacterial colonies.* F. magna* was identified by gram stain and biochemical reactions on the RapID Ana II (Innovative Diagnostic Systems, Inc., Atlanta, GA) identification strip.

CT abdomen and pelvis as well as an echocardiogram were performed to look for a potential source for hematogenous spread of infection to the wrist. These tests were normal suggesting that the infection was acquired through direct inoculation of wound at the time of injury and/or contamination of the wound with oral saliva applied on the wound by the patient.

Antibiotic therapy was deescalated to IV Clindamycin 900 mg Q8H once fluid cultures from the CT guided aspiration of the wrist joint grew* F. magna*. The patient was treated with a total of nine weeks of Clindamycin which is comprised of Clindamycin 900 mg intravenously Q8H for the first two weeks followed by oral Clindamycin 300 mg Q6H for the next seven weeks. This high dose and extended duration of therapy for 9 weeks, as opposed to the conventional therapy of 4 weeks for native joint septic arthritis, was undertaken because of the patients persistently elevated inflammatory marker (C-reactive protein of 23.87) and residual clinical examination findings of a low grade wrist swelling and erythema at the time of discharge and during subsequent outpatient followup visits. All of these resolved completely by extending the antibiotic therapy beyond the stipulated conventional time frame based upon ongoing clinical symptomatology, physical examination findings, and laboratory markers such as CRP.

After completion of antibiotic therapy the subsequent outpatient orthopedic and primary care visits indicated that the right wrist swelling, tenderness, warmth, and erythema resolved completely. The incisions healed completely. The surgical scar was healthy and well approximated. A functional wrist brace was prescribed. No recurrent infections occurred. No osteosynthetic wrist implants were required. There was residual pain of low intensity upon active movements. He did have a mild flexion contracture. He had mildly restricted movements in regard to his dorsiflexion, volar flexion, and rotation of the wrist. His power was about 50% less than the opposite hand when attempting to make a fist. He was able to write with some effort and able to oppose his thumb. To improvise on these residual deficits Naprosyn and physical therapy were prescribed and he was subsequently able to return to work with minor restrictions on lifting weights and performing strenuous activity with his wrist. He became occupationally functional after approximately 2.5 months from the time of his initial presentation.

## 3. Discussion

Septic arthritis is a relatively common rheumatologic condition. Anaerobic bacteria account for 20% of these infections with* F. magna* (previously known as* Peptococcus magnus* or* Peptostreptococcus magnus*) and* Peptostreptococcus prevotii* being the predominant isolates.* F. magna* has been described as one of the most frequent pathogens in the etiology of postoperative and prosthetic implant associated septic arthritis [1–3]. Fewer reports exist of such infections in a previously normal joint or a joint with secondary osteoarthritis from prior closed trauma.

*F. magna* predominantly inhabits the skin as well as the mucosa of gastrointestinal and urogenital tracts as a normal commensal [4]. It is identified in 5–12% of all anaerobic infections and accounts for 20–40% of all gram positive anaerobic cocci (GPAC) infections [5, 6]. Being a slow grower with predilection towards developing resistance to antimicrobials, infection with this organism results in an arduous course of disease and protracted treatment. Of interest,* F. magna*—despite being described as a common etiologic agent in bone infections—was disregarded for a number of years following the publication of an article which described the expensive and fastidious nature of the anaerobic culture methodology for these bacteria [7]. In recent times, with the advances in isolation and culture techniques, the ability to identify anaerobic infections has dramatically improved.

We reported an infection in a small joint—the wrist. A complete review of the literature revealed one report of* F. magna* infection involving a wrist joint [8]; however most reports describe* F. magna* infections to involve larger joints [9].

Descriptions of anaerobic septic arthritis usually suggest involvement by a single organism [9]. Our patient's presentation was delayed despite of the presence of an open wound. The open wound predisposed him to a polymicrobial infection. On the contrary, a pure culture of* F. magna* was identified in our patient. The open wound may have served as the port of entry for the infection.

*F. magna* in chronic wounds impairs the process of wound healing [10]. Pathophysiologic mechanisms involved in such infections are a variety of bacterial virulence factor that this organism expresses [11]—binding of significant amounts of human serum albumin to a specific protein receptor on the cell wall of the organism, production of proteolytic enzymes by the bacteria, and expression of a surface protein, Protein L, which binds to the *κ* light-chain variable region of the human immunoglobulin molecule initiating the release of inflammatory mediators.

Numerous treatment options are available to treat* F. magna* infections including Metronidazole, Linezolid, Tigecycline, Carbapenems, and Piperacillin/Tazobactam with variable susceptibility to Penicillin and Clindamycin [12, 13]. Our patient responded well to treatment with Clindamycin over a prolonged course to ensure complete resolution of clinical signs and symptoms of infection as well as normalization of laboratory inflammatory markers. A narrower spectrum agent like Amoxicillin or Ampicillin was not chosen on account of its poor bone penetrance. Clindamycin has excellent bone penetrance and therefore offers the highest chances of salvaging the wrist joint by ensuring complete eradication of the infectious agent from the joint. This approach proved “limb saving” in our case. Increasing evidence suggests decreased susceptibility of* F. magna* to Erythromycin and Tetracycline [14].

In conclusion, infections with* F. magna* should be considered in the differential diagnosis when evaluating cases of septic arthritis. Given the potentially increased reporting of infections with* F. magna*, considering it a mere contaminant would no longer be advisable.
